# Isolation and Characterization of the Zearalenone-Degrading Strain, *Bacillus spizizenii* B73, Inspired by Esterase Activity

**DOI:** 10.3390/toxins15080488

**Published:** 2023-08-02

**Authors:** Xue Liu, Na Wu, Mingyu Zhang, Feng Xue, Qing Xu

**Affiliations:** 1 School of Food Science and Pharmaceutical Engineering, Nanjing Normal University, Nanjing 210046, China; 2 College of Marine and Bioengineering, Yancheng Institute of Technology, Yancheng 224007, China

**Keywords:** zearalenone, *Bacillus spizizenii*, detoxification, cell extract, enzyme

## Abstract

Zearalenone (ZEN) is a widespread mycotoxin found in grain and feed, presenting a serious threat to animal and human health. This study investigated the ability of the novel strain B73, isolated from petroleum-contaminated soil, to detoxify ZEN. B73 was identified as *Bacillus spizizenii* through physiological and biochemical tests, and further confirmed based on the 16S rRNA gene sequence and the complete genome sequence. *B. spizizenii* B73 was capable of degrading up to 99.3% of ZEN at a concentration of 10 μg/mL in a minimal medium (pH = 7.0) within 8 h at 37 °C via HPLC-UV. In addition, *B. spizizenii* B73 was used to treat ZEN-contaminated wheat bran, dried distillers grains (DDGS), and corn meal, whereby the respective degradation rates reached 96.32%, 98.73%, and 80.31% after 36 h of treatment. HPLC-Q-Exactive-MS/MS analysis revealed one of the degradation products to have the formula C_17_H_24_O_4_. *B. spizizenii* B73 is a novel strain isolated from petroleum-contaminated soil, and the extracellular enzymes secreted by this strain show a remarkable ability to degrade ZEN.

## 1. Introduction

Zearalenone (ZEN; 6-(10-hydroxy-6-oxo-trans-1-undecenyl)-bresorcylic acid lactone) is a non-steroidal estrogenic mycotoxin that is synthesized by several species of fungi, including *Fusarium graminearum*, *Fusarium culmorum*, *Fusarium equiseti*, and *Fusarium verticillioides* [1,2]. As a natural estrogen, ZEN has the ability to bind to estrogen receptors found in mammals, potentially resulting in reproductive disorders in livestock [3]. Moreover, extensive research has demonstrated that ZEN is carcinogenic and may induce hematotoxicity, immunotoxicity, and genotoxicity [4,5]. Consequently, ZEN-contaminated products pose a significant risk to both livestock and human health. ZEN is a highly prevalent mycotoxin found in moldy wheat, corn, and other cereal crops, and is considered one of the most pervasive mycotoxins worldwide [6]. Some types of grains, including corn, are highly susceptible to contamination by ZEN throughout the planting, harvesting, and storage processes [7]. Currently, strict guidelines for the maximum content of ZEN are implemented in several countries. For instance, the European Union (EU) has set a specification that refined grains must not contain more than 100 µg/kg of ZEN [8]. Hence, there is a pressing need to eliminate ZEN from grains. Based on the physical and chemical characteristics of ZEN, its elimination techniques mainly encompass physical detoxification, chemical degradation, and biological degradation.

Among the various ZEN degradation methods, biological detoxification has emerged as a promising strategy, owing to its impressive degradation efficiency, specificity, and minimal environmental impact [9]. Intracellular or extracellular enzymes secreted by microorganisms have the ability to metabolize ZEN into non-toxic or low-toxicity compounds [10]. Microorganisms commonly used in ZEN biodegradation include bacteria, fungi, and molds. Certain bacteria and fungi, including *Bacillus*, *Lactobacillus plantarum*, and *Rhizopus*, were found to degrade ZEN [11,12,13]. For instance, Pereyra et al. conducted an evaluation on the impact of 11 strains on ZEN, whereby all strains demonstrated the ability to degrade 58–96.9% of ZEN within a time frame of 72 h [14]. Vekiru et al. demonstrated that *Trichosporon mycotoxinivorans* could degrade 95% of ZEN within 48 h. Furthermore, these researchers discovered that the lactone ring of ZEN was hydrolyzed into non-toxic by-products [15]. *Aspergillus niger* FS10 was found to secrete ZEN-degrading extracellular enzymes into the fermentation broth, which allowed it to degrade 90% of the ZEN within 8 days [16]. In addition, some strains were observed to degrade ZEN in grains. For instance, Chen et al. demonstrated that the *Bacillus* strain B2 was capable of degrading 56% of 5 mg·kg^−1^ of ZEN in maize after 72 h [17]. In a solid-state fermentation study, Cho et al. found that 95% of 0.25 mg·kg^−1^ ZEN could be degraded within 48 h [18]. Moreover, *Bacillus megaterium* BM344-1 was capable of degrading 25% of 1.5 μg/mL of ZEN within 44 h in maize [19]. Despite their potential, the efficiency of these microorganisms in degrading ZEN is limited by their prolonged degradation period, thus highlighting the importance of screening strains with a rapid degradation ability. Furthermore, the degradation mechanisms of many strains remain unclear, and the toxicity of degradation products is yet to be determined, which limits their application potential.

In this study, we assessed the efficacy of the newly screened strain B73 in degrading ZEN, and its potential application for degrading ZEN in grains. The 16S rRNA sequencing analysis confirmed the strain as belonging to *Bacillus spizizenii*. Semi-solid fermentation was employed to treat ZEN-contaminated corn cobs, and the main metabolite secreted by B73 during ZEN degradation was evaluated using high-performance liquid chromatography tandem quadrupole–electrostatic field orbitrap high-resolution mass spectrometry (HPLC-Q-Exactive-MS/MS) analysis. Our study contributes a novel strain that is highly efficient in degrading zearalenone.

## 2. Results 

### 2.1. Isolation, Screening, and Identification of ZEN-Degrading Bacteria

**Figure 1:**
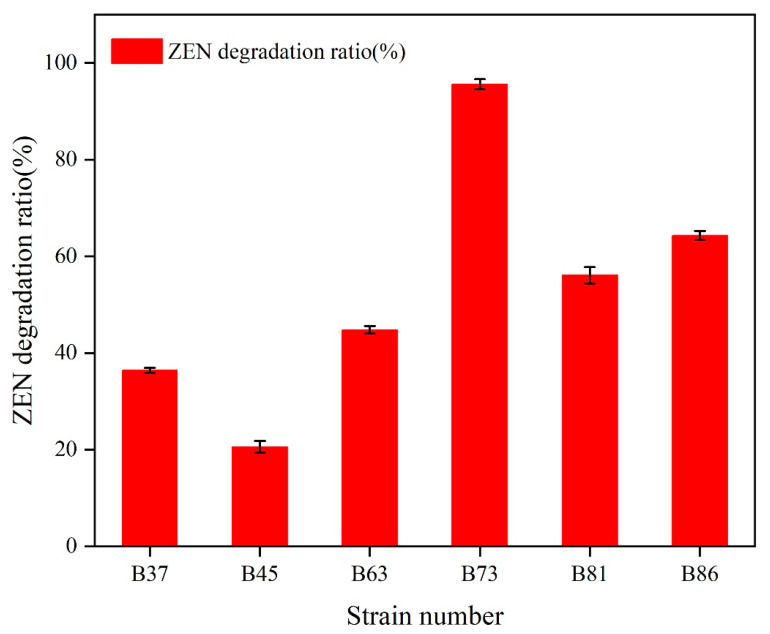
The ZEN degradation rate in MM medium by different strains. Data represent the mean ± SD of three independent replicates.

The strains were isolated from soil contaminated with petroleum using ZEN as the sole carbon source. Six strains, named B37, B45, B63, B73, B81, and B86, were found to be capable of degrading ZEN after three successive rounds of enrichment. Among them, strain B73 demonstrated remarkably efficient ZEN degradation, with a degradation rate of more than 95.6% after 24 h of incubation with 10 μg/mL ZEN in a minimal medium (MM) at 37 °C (Figure 1).

**Figure 2:**
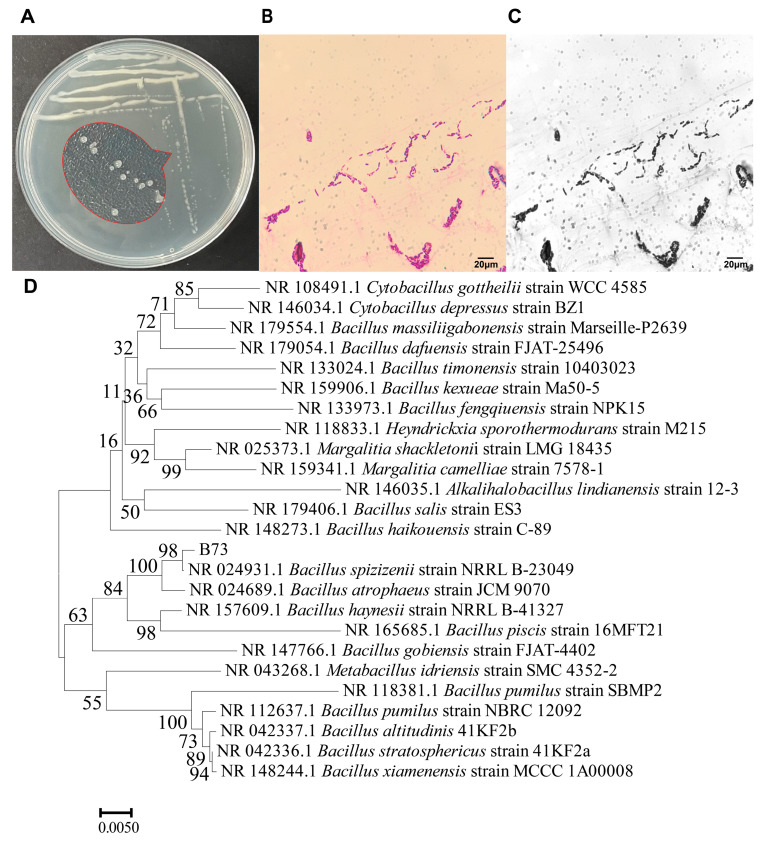
Colony morphology (**A**) and cell morphology (**B**,**C**) of strain B73; (**D**) Phylogenetic tree of strain B73 based on 16S rDNA gene sequence.

To visualize the morphology of B73, we densely inoculated the strain on Luria–Bertani (LB) agar medium. As illustrated in Figure 2A, the colonies of B73 were transparent, viscous, convex and circular with smooth edges. Upon Gram staining, the cells of B73 exhibited a rod-shaped morphology with a purple color, indicating that it was a Gram-positive *bacillus* (Figure 2B,C) and possibly a member of the eponymous genus [20].

The 16S rDNA gene sequence of strain B73 was analyzed by conducting a BLAST search the National Center for Biotechnology Information (NCBI) GenBank database (https://www.ncbi.nlm.nih.gov/, (accessed on 15 April 2023)), which revealed 98% sequence similarity to *Bacillus spizizenii* (GenBank ID in NCBI: NR 024931.1). Then, a phylogenetic tree was constructed based on 24 16S rDNA sequences with high homology, using the maximum likelihood (ML) model with 500 bootstrap replicates and the neighbor-joining (NJ) method in MEGA X [21]. As shown in Figure 2D, strain B73 exhibited the highest similarity to *Bacillus spizizenii* NRRL B-23049 (GenBank ID: NR027552.1). After conducting a series of phenotypic experiments (colony morphology observations on agar plates and microscopic observations under a microscope) and sequencing of the 16S rDNA gene, it was determined that the ZEN-degrading isolate was indeed a novel strain of *Bacillus spizizenii*.

### 2.2. Degradation of ZEN via B73

**Figure 3:**
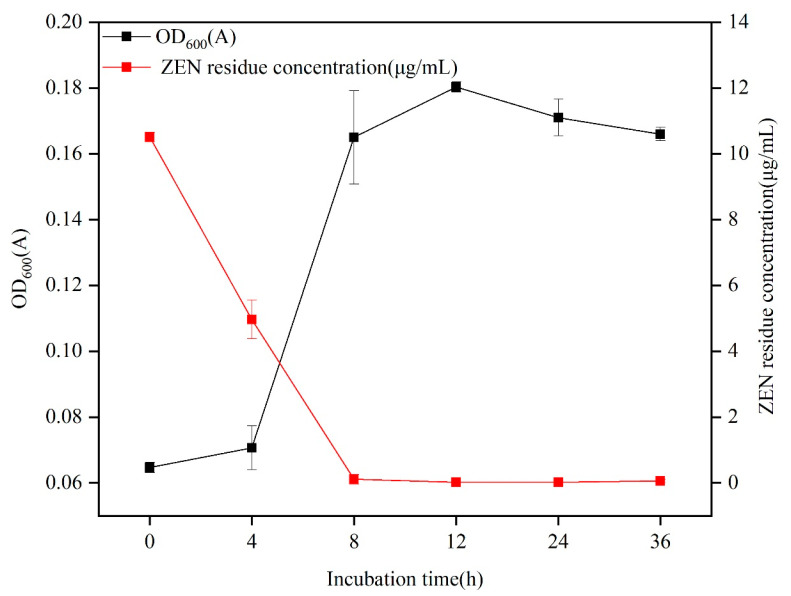
Strain B73 growth curve and ZEN degradation ratio. Data represent the mean ± SD of three independent replicates.

As shown in Figure 3, *B. spizizenii* B73 demonstrated a ZEN degradation efficacy of 99.3% in a minimal medium after 8 h of incubation. Following optimization of pH, temperature, inoculation amount, and ZEN concentration (Supplementary Materials: Figure S1), the ZEN residue was only 0.07 μg/mL after 8 h at 37 °C. Furthermore, the strain B73 exhibited rapid growth during ZEN degradation, and entered the stationary phase after 12 h. The slower growth of strain B73 after 8 h could be attributed to the depletion of ZEN as the sole carbon source in the minimal medium. The degradation of ZEN by B73 was primarily observed during the initial phase of rapid growth, indicating a strong correlation between the growth metabolism of strain B73 and the breakdown of ZEN. 

### 2.3. Degradation of ZEN by Different Fractions of the B73 Culture Broth

**Figure 4:**
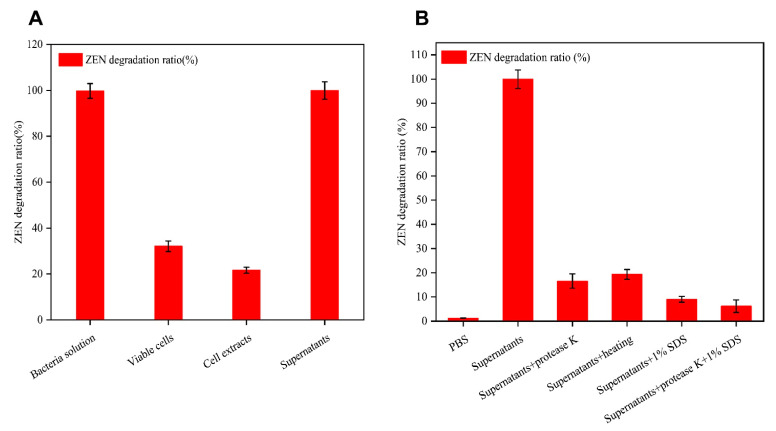
(**A**) Removal of ZEN by different fractions of strain B73; (**B**) ZEN degradation of cell-free supernatant after heating; SDS and proteinase K treatment. Data represent the mean ± SD of three independent replicates.

To investigate the mechanism by which B73 degraded ZEN, we compared the rates of ZEN degradation via different fractions of the B73 culture, including the cell extract, cell-free supernatant, culture broth, and washed viable cells. As shown in Figure 4A, the cell-free supernatants exhibited the highest ZEN degradation rate, reaching up to 99.92% at the 8 h. This value was similar to the untreated culture broth, which achieved a degradation rate of 99.73%. By contrast, the degradation rates of the cell extract and washed viable cells were 21.68% and 32.1%, respectively. It should be noted that the reduction in ZEN content by living cells may be related to passive absorption to the glucan of the cell wall [22]. We thus speculated that extracellular enzymes secreted by B73 play a critical role in ZEN degradation. 

To further verify whether enzymes in the cell-free supernatants play a key role in ZEN degradation, we subjected the supernatants to protease K treatment with and without sodium dodecyl sulfate (SDS) prior to adding ZEN to the mixture. SDS can denature most proteins, while protease K degrades the polypeptide chain, potentially reducing the degradation of ZEN [23]. As shown in Figure 4B, the combination of protease K and SDS yielded the lowest degradation rate of ZEN in the cell-free supernatants, degrading it by 6.19%. By contrast, when either protease K or SDS was used alone, the degradation rates reached only 16.5% and 9.0%, respectively. In addition, after heat treatment of the cell-free supernatant at 100 °C for 10 min, the degradation rate of ZEN reached only 19.3% after 8 h of incubation. Prior research suggested that high temperatures can denature active proteins, thus reducing the efficiency of ZEN degradation [24,25]. Based on our findings, it is likely that B73 may produce a hydrolase enzyme that is responsible for the observed degradation of ZEN [26]. In addition, Pan et al. showed that the reductase secreted by *Candida parapsilosis* ATCC 7330 could convert ZEN into less toxic beta-zearalenol (beta-ZOL) [27]. Although our results indicate that the extracellular enzyme produced by strain B73 is capable of degrading ZEN, further analysis and research are required to fully understand the specific degradation mechanism involved.

### 2.4. Exploration and Analysis of ZEN Degradation Products

**Figure 5:**
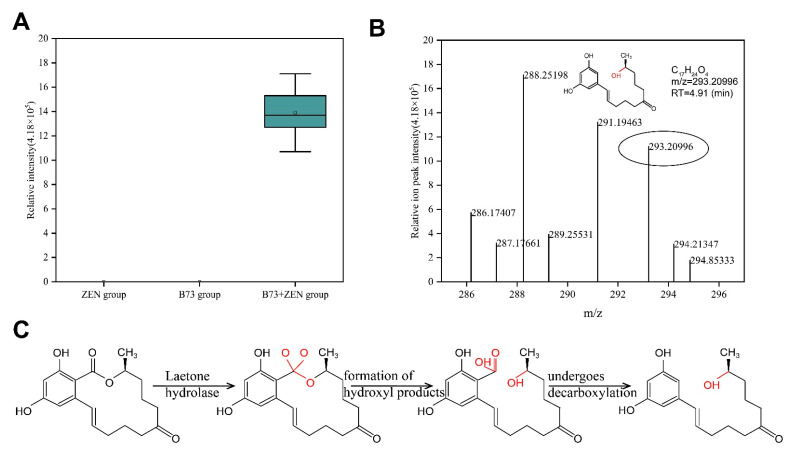
(**A**) Analysis of degradation product BP-1 content; (**B**) structure analysis of degradation products; (**C**) molecular structure transformation path. Data represent the mean ± SD of three independent replicates.

To further explore the ZEN degradation mechanism of extracellular enzymes produced by strain B73, we employed UPLC-Q-Exactive-MS to analyze the degradation products. Our analysis detected a potential degradation product in positive ion mode, which we named BP-1 (Figure 5A). BP-1 was not detected in samples that only contained ZEN or B73, thus confirming that the substance did not originate from these samples. However, BP-1 was found in samples of B73 + ZEN, suggesting that BP-1 was produced through the degradation of ZEN by B73 (Figure 5A). BP-1 has a predicted molecular weight of 292 g/mol and its mass spectrum is consistent with the molecular formula of C_17_H_24_O_4_ (Figure 5B). The process by which lactone hydrolase degrades ZEN and produces degradation products follows a similar pattern [28]. We hypothesize that B73 degrades ZEN mainly through lactone hydrolase activity, which converts the C=O group at C11 of the oxygen-containing lactone ring of ZEN into two C-O groups. This leads to the original C-O bond breaking and the formation of a new C=O bond, which spontaneously undergoes decarboxylation to form the non-estrogenic degradation product BP-1 (Figure 5C). Thus, the BP-1 product obtained in our study is most likely the result of lactone hydrolase activity.

### 2.5. ZEN Degradation by B73 during Semisolid Fermentation of Grain Products

**Figure 6:**
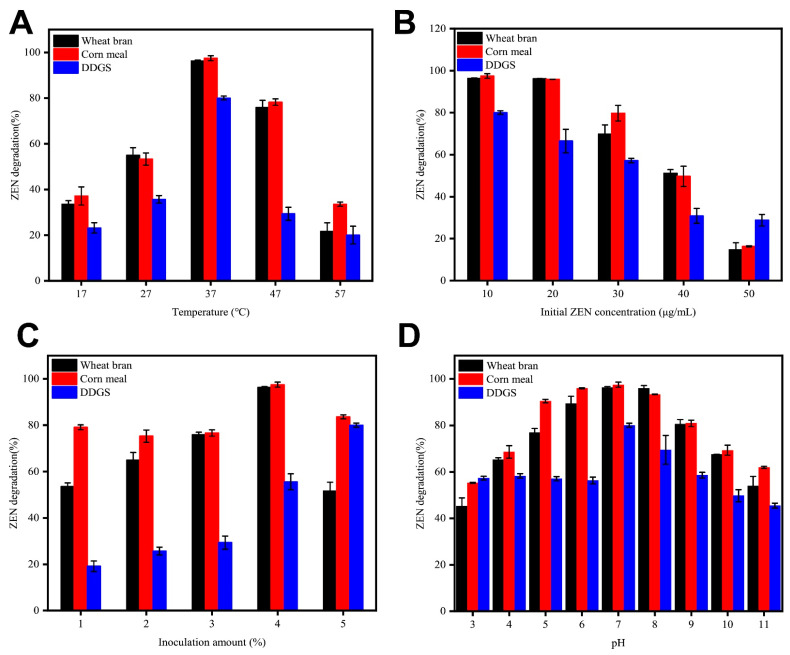
Effect of the medium conditions for strain B73 on ZEN degradation in wheat bran, corn meal and DDGS: (**A**) temperature, (**B**) initial ZEN concentration, (**C**) inoculation amount, and (**D**) pH. Data represent the mean ± SD of three independent replicates.

To explore the possible utilization of B73, we conducted a study on the degradation of ZEN using B73 in a semisolid mixture of grain products, including wheat bran, corn meal, and dried distiller’s grains (DDGS). Initially, we evaluated the efficacy of ZEN degradation by different strains under varying cultivation conditions and subsequently optimized the degradation conditions of B73. This optimization process involved fine-tuning factors, including temperature, inoculum size, initial ZEN concentration, and pH value, to enhance the efficiency of ZEN degradation. The degradation kinetics of ZEN were observed over a period of 36 h (Figure 6). 

Figure 6A shows the effect of incubation temperature on ZEN degradation by B73. The results indicate that B73 effectively degraded ZEN in three semisolid grain products at temperatures ranging from 17 to 57 °C. The degradation efficiency of ZEN increased with increasing temperatures up to 37 °C, but the rate of ZEN degradation gradually decreased at higher temperatures. The optimal temperature for ZEN degradation was observed to be 37 °C, based on the peak degradation rate of B73. At this temperature, the values for ZEN degradation in wheat bran, corn meal, and DDGS reached 95.7%, 98.4%, and 78.94%, respectively.

Previous studies demonstrated that ZEN has the capacity to impede DNA and protein synthesis, potentially impacting the growth and viability of the strain [29]. Therefore, we examined the effects of various initial concentrations of ZEN on its degradation by B73 (Figure 6B). Our findings demonstrate that B73 was able to efficiently degrade ZEN at initial doses ranging from 10 to 30 μg/mL. However, at concentrations greater than 40 μg/mL, the ZEN degradation performance of B73 was significantly reduced. This could be attributed to the toxic effects of high ZEN concentrations, which gradually inhibited the growth and degradation ability of B73. The maximum degradation rate of ZEN by B73 was observed at an initial concentration of 10 μg/mL, indicating that this concentration is optimal for degradation. Our findings demonstrate that wheat bran, corn meal, and DDGS achieved degradation rates of 96.3%, 97.4%, and 80.04%, respectively.

The effect of varying amounts of B73 inoculum on ZEN degradation rate is shown in Figure 6C. The results revealed that the degradation rate increased with increasing inoculum amounts in the range of 1–4%. This can be explained by the fact that a lower amount of B73 inoculum extended the lag phase, reducing the final biomass and overall degradation effect of B73. However, a slight decrease in the degradation rate of ZEN was observed when the inoculation amount of B73 was further increased from 4 to 5%, suggesting that B73 may not be able to fully utilize the carbon source for growth at high inoculum levels. The optimal inoculation amount for strain B73 was identified to be 4%, which achieved impressive degradation rates of 96.3%, 98.0%, and 80.27% in wheat bran, corn meal, and DDGS samples containing 10 mg/kg ZEN, respectively.

Finally, we investigated the impact of pH on ZEN degradation by B73. Our findings demonstrate that B73 exhibited highly efficient ZEN degradation across a broad range of pH levels, from 3.0 to 11.0 (Figure 6D). Nevertheless, the highest rates of ZEN degradation were observed at a pH of 7.0, reaching 96.25%, 97.2%, and 79.6% in wheat bran, corn meal, and DDGS, respectively.

Based on the above analysis, we determined the optimal degradation conditions for ZEN, including pH 7.0, temperature 37 °C, and concentration 10 μg/mL, with a vaccination dose of 4%. Under these conditions, the degradation rates of B73 in wheat bran, corn flour, and DDGS were 96.32%, 98.73% and 80.31%, respectively.

## 3. Discussion

In this study, we investigated numerous strains selected for their ability to degrade ZEN, and identified the highly efficient strain, B73. As shown in Figure 2, the morphology of B73 and sequencing results confirm its classification as a strain of *Bacillus spizizenii*. Furthermore, based on the *Bacillus subtilis* taxonomy established by Yi et al., we can infer that *Bacillus spizizenii* is a subspecies of *Bacillus subtilis* [30]. *Bacillus subtilis* is utilized as a probiotic due to its exceptional stability in various environmental conditions [31]. Probiotics have been found to play a significant role in regulating the gut microbiome, which helps to balance inflammation and promote animal growth. Probiotics are commonly added to food and grains, as well as being used the fermentation process of soybeans and production of kimchi [32]. Studies have demonstrated that the consumption of *Bacillus spizizenii* can yield health benefits as it is capable of withstanding acidic conditions in the stomach and surviving in the intestinal tract [33]. When added to soybean grains, *Bacillus subtilis* has the potential to enhance the production of hypoallergenic feed, which is suitable for newly weaned piglets or calves [34]. Furthermore, research has demonstrated that the antimicrobial peptide (AMP) extracted from *Bacillus spizizenii* exhibits inhibitory effects against *Staphylococcus aureus*, and thus, has the potential to function as a substitute for antibiotics in animal feed [35]. Based on these findings, *Bacillus spizizenii* is considered to be a probiotic with a high level of safety when employed for ZEN elimination from grains.

Recently, several strains of probiotics have been found to be effective in degrading ZEN. Notably, lactic acid bacteria (LAB) are promising biological agents for combating *Fusarium* and reducing ZEN levels in grains by up to 23% [36]. The probiotic strain *B. amyloliquefaciens*, which was isolated from moldy corn samples, was capable of degrading 5 μg/mL of ZEN within 36 h. Additionally, this strain exhibited notable acid and drug resistance [37]. Furthermore, previous research has established that the *Bacillus licheniformis* strain CK1 exhibits probiotic characteristics, including resistance to acidic conditions and the inhibition of pathogens. Moreover, this particular strain has demonstrated the capability of degrading 5 μg/mL ZEN by 73% in 24 h [38]. Francis et al. reported that *K. pneumoniae* GS7-1 completely degraded 5 μg/L ZEN within 96 h [39]. Wu et al. previously demonstrated that *Stappia* sp. WLB29 could effectively degrade 10 μg/L ZEN in minimal medium, achieving a degradation rate of 98.8% after 60 h [40]. However, these strains typically require considerable time to degrade ZEN and are only effective at low ZEN concentrations. In this study, we aimed to investigate the degradation ability of probiotic B73 towards ZEN in an MM, as well as the degradation ability of the strain under varying conditions such as different temperatures, pH levels, inoculation amounts, and varying concentrations of ZEN. In our study, B73 exhibited similarly efficient ZEN degradation in both an MM and grains. In particular, it was found that B73 exhibited a superior degradation ability when grown in an MM, as it was able to degrade 99.3% of 10 μg/mL ZEN after only 8 h (Figure 3). Moreover, this strain demonstrated a remarkable adaptability to a diverse range of conditions during semi-solid fermentation, including higher ZEN concentrations as well as wider pH and temperature ranges than comparable strains. However, it should be noted that the degradation rate of ZEN decreased as the pH of the culture medium and incubation temperature increased or decreased. These findings are in line with those reported in previous studies [41]. Currently, there is a lack of studies on the rapid and effective degradation of concentrated ZEN in grains using semi-solid fermentation. Existing research suggests that *Bacillus* has the ability to form endospores under stressful conditions such as extreme temperatures, acidity and alkalinity, enabling it to survive in hostile environments [42]. In comparison to other probiotics, *Bacillus spizizenii* is active at an extensive range of ZEN concentrations and pH levels, making it a favorable choice for practical applications in agriculture and industry. 

While significant progress has been made in the analysis of key enzymes and mechanisms involved in the biodegradation of ZEN, the potential for unknown adverse effects or high levels of toxicity from estrogenic products has hindered the further application of the microbial degradation of ZEN in actual production. In one study, Yi et al. isolated *Bacillus licheniformis* CK1 and observed the activities of xylanase, cellulase and protease in the culture supernatant, suggesting that CK1 may improve the digestibility of dietary nutrients. However, a further toxicity analysis of degradation products was not conducted [43]. During the process of ZEN degradation, numerous derivatives are generated, including zearalenone 14-glucoside (Z14G) and zearalenone 14-sulfuric acid (Z14S), both of which have low estrogenic activity [44]. Mokoena et al. reported 68% and 75% reductions in ZEN content in the medium after 4 days of culturing with lactic acid bacteria. However, the toxicity of the product remained unchanged in the SMO human esophageal cancer cell line. Therefore, it was deemed an ineffective method of detoxification [45]. In this study, we analyzed the degradation product BP-1 in positive ion mode (*m*/*z* = 293.20996 [M + H]^+^). Previous research conducted by Wang et al. also utilized a positive ion mode (*m*/*z* = 293.1743 [M + H]^+^) and estimated the relative molecular weight of the degradation product to be 292 [46]. We therefore postulated that the degradation product of B73 had a relative molecular weight of 292 and the molecular formula of C_17_H_24_O_4_. The obtained degradation product exhibited similarities to the lactone hydrolase product reported by Zhou et al. [47]. The lactone bond in the ZEN structure has been identified as the primary source of toxicity. Recent studies indicate that lactone hydrolases can disrupt the lactone bond of ZEN, resulting in the opening of the ring structure and formation of a straight-chain product, which subsequently undergoes decarboxylation. The resulting decarboxylation products do not bind to proteins in the endoplasmic reticulum, resulting in a significant reduction or even elimination of ZEN toxicity [48]. However, further research is necessary to elucidate the specific degradation mechanism and identify potential degradation targets.

The degradation of ZEN via *Bacillus spizizenii* B73 in both liquid culture and actual grains is significantly affected by environmental conditions, including temperature, pH, inoculation amount, and ZEN concentration. The identification of optimal conditions can provide valuable insights for the development of efficient ZEN control strategies in grains. Further exploration of extracellular enzymes can enhance the rate of ZEN degradation.

## 4. Materials and Methods

### 4.1. Reagents and Culture Media

The ZEN standard (the CAS number: Z299705) was obtained from Shanghai Aladdin Biochemical Technology Co., Ltd. (Shanghai, China) and was dissolved in chromatographic-grade acetonitrile to a concentration of 25 g/L to prepare the stock solution. HPLC-grade acetonitrile and methanol were procured from Sigma-Aldrich (St. Louis, MO, USA). For the degradation experiment, the initial solution was supplemented in the reaction system to achieve the indicated final concentration. The bacterial strain was cultured in LB medium comprising 1% peptone, 0.5% yeast extract, and 1% NaCl, for 12 h at 37 °C and 200 rpm, after which it was transferred at a 1% inoculation ratio to MM, comprising 1.52 g KH_2_PO_4_, 2.44 g Na_2_HPO_4_, 0.2 g MgSO_4_·7H_2_O, 0.5 g (NH_4_)_2_SO_4_, and 0.05 g CaCl_2_, as well as 10 μg/mL ZEN as the sole carbon source.

### 4.2. Screening and Isolation of Bacillus Strains

The soil samples were added to a 250 mL shaking flask and placed in a shaking incubator (Shanghai Minquan Instrument Co., Ltd., Shanghai, China) for uniform vibration. Specifically, samples comprising 2.5 g of soil were suspended in 25 mL of sterile phosphate-buffered saline (PBS) with a pH of 7.4, shaken at 200 rpm for 30 min at room temperature, and allowed to settle for 10 min. Next, the supernatant was serially diluted in sterile PBS (10^−1^ to 10^−9^), and 300 µL of each dilution was used to inoculate 30 mL of MM supplemented with 10 μg/mL ZEN, followed by incubation in a shaking incubator (Shanghai Minquan Instrument Co., Ltd., Shanghai, China) at 37 °C and 200 rpm for 24 h. Subsequently, 1 μL of the resulting culture was spread on MM agar plates containing 10 μg/mL ZEN and incubated at 37 °C until single colonies developed. The single colonies were then streaked on LB agar plates three times to obtain pure strains. The best strain, *Bacillus subtilis* subsp. *spizizenii* B73, was deposited at China General Microbial Culture Collection under CGMCC No. 22957. 

### 4.3. Identification of Bacillus Strains 

The B73 strain was identified via sequencing the 16S rDNA gene. Bacterial genomic DNA extraction was performed using the Bacterial Genomic DNA Extraction Kit (Solarbio) following the manufacturer’s instructions. The 16S rDNA sequence was amplified using the universal primers 27F: 5′-AGAGTTTGATCCTGGCTCAG and 1492R: 3′-GGTTACCTTAGGACTT in Sangon Biotech (Shanghai, China) Co., Ltd. The PCR reaction conditions were as follows: initial hot start at 95 °C for 5 min, followed by 26 cycles of denaturation at 95 °C for 30 s, annealing at 56 °C for 30 s, and extension at 72 °C for 90 s, and a final extension step at 72 °C for 10 min. The PCR products were sequenced by Nanjing Kinco Biotechnology Limited Company, and the obtained 16S rDNA sequence was analyzed by performing a BLAST search against the NCBI database. A phylogenetic tree was constructed using MEGA software.

### 4.4. Whole-Genome Sequencing

Based on a previously reported method [20], the genomic DNA of strain B73 was extracted using the E.Z.N.A. Bacterial DNA Kit (Omega Bio-Tek Inc., Norcross, GA, USA). The Illumina Hiseq×10 platform, which is a widely used second-generation sequencing platform, was utilized to construct fragments with insertion of ~400 bp from the qualified DNA samples for paired-end sequencing. The raw sequencing data were obtained from each sample with a coverage depth of no less than 100×. Multiple genomes were then assembled using scaffolds.

### 4.5. Effect of Culture Conditions on the Degradation of ZEN

After being activated at 37 °C for 12 h, a 10 µL aliquot of B73 culture was used to inoculate DF medium. All experimental groups, except for the one designed to test the inoculation dosage, received an inoculum of 1% in DF medium that contained 10 μg/mL ZEN. The culture was then incubated at 37 °C, with shaking at 200 rpm, and samples were collected at regular intervals to monitor the degradation of ZEN by B73. The biomass of each sample was determined by measuring its optical density at 600 nm (OD_600_), while the residual amount of ZEN was quantified using HPLC (Agilent 1200, UV detector, Hercules, CA, USA).

A number of culture conditions were tested, including the inoculation amount (1%, 2%, 3%, 4%, 5%), temperature (ranging from 17 °C to 57 °C), initial pH (from 3.0 to 11.0), and varied initial ZEN concentrations (from 10 μg/mL to 50 μg/mL). The cultures were incubated at 200 rpm and 37 °C. Samples were collected at 0, 4, 8, 12, 24 and 36 h of fermentation and inactivated via the addition of an equivalent volume of cold methanol. Subsequently, the samples were subjected to HPLC analysis to determine the degradation rate. An MM that contains the same concentration of ZEN without bacteria was included as a control. Each set of culture conditions was replicated thrice.

### 4.6. ZEN Degradation by Different Fractions of the Culture Broth

Based on the methodologies employed by Rao et al. and Zhai et al. [49,50], this study aimed to evaluate the efficacy of various fractions of B73 culture broth, including the cell-free supernatant, washed viable cells, cell extract, and bacteria solution. The B73 strain was cultivated in LB medium at 37 °C and 200 rpm for 12 h. The culture broth was collected as “bacteria solution”. In addition, the culture broth was subjected to centrifugation at 6000 rpm for 8 min, yielding the supernatant and cell pellet as separate fractions. The supernatant was filtered using a 0.22 µm sterile filter membrane and designated as “cell-free supernatant”. The cell pellet was resuspended in an appropriate volume of phosphate buffer (pH 7.4) and labeled as “viable cells”. The viable cells were subjected to sonication at 360 W for 20 min (interval 5 s), followed by filtration using a 0.22 µm sterile filter membrane and labeling as the “cell extract”. ZEN at a concentration of 10 µg/mL was added to the fractions, followed by incubation at 37 °C and 200 rpm to evaluate the degradation of ZEN in each fraction.

### 4.7. Characterization of the Active ZEN-Degrading Component in a Cell-Free Supernatant

To investigate whether ZEN degradation was enzyme-dependent, the cell-free supernatant was subjected to a heat treatment at 100 °C for 10 min. Additionally, the samples were exposed to sodium dodecyl benzene sulfonate (SDS), proteinase K (1 g/L), and a combination of proteinase K plus SDS to assess the degradation of ZEN, as described by Alberts et al. [51]. The samples were incubated at 37 °C for 12 h, with PBS as a negative control.

### 4.8. Degradation of ZEN by B73 in Wheat Bran, DDGS and Corn Meal 

To investigate the effectiveness of B73 in degrading ZEN in grain products, degradation experiments were conducted using wheat bran, dried distiller’s grains (DDGS), and corn meal. With the exception of the inoculation dose experiment, all experimental groups used a 1% inoculation dose of strain B73 in MM medium, which contained 10 μg/mL ZEN, and had a content of 50% wheat bran, corn meal, or DDGS. To study the impact of different culture conditions on the ZEN degradation efficiency, we varied the inoculation amount (1%, 2%, 3%, 4%, 5%), temperature (17 °C, 27 °C, 37 °C, 47 °C, 57 °C), initial pH (ranging from 3.0 to 11.0), and initial ZEN concentration (ranging from 10 μg/mL to 50 μg/mL). All cultures were incubated at 37 °C and 200 rpm. After 72 h of culture, samples were collected and the reaction was terminated by adding a 10-fold volume of cold methanol, followed by high-performance liquid chromatography (HPLC) analysis of the ZEN content to determine the degradation rate. A control mixture comprising the same concentration of ZEN and grain products but excluding strain B73 was included as a negative control. Each set of culture conditions was replicated three times.

### 4.9. Exploration and Analysis of ZEN Degradation Products

Strain B73 was cultured in the presence of ZEN at 37 °C and 200 rpm. Samples were collected at 0, 12, 24, and 36 h, and the reactions were terminated by adding an equal volume of methanol. The sample at 0 h served as the blank control group. Subsequently, the samples were subjected to identification and analysis using HPLC-Q-Exactive-MS. Among them, HPLC-Q-Exactive-MS/MS consisted of an Ulti Mate 3000 high-performance liquid chromatography system (Diane, USA) and Q-Exactive-MS/MS high-resolution mass spectrometer (Thermo Fisher Scientific, Waltham, MA, USA). A Phenomenex Kinetex XB C18 column (4.6 mm × 100 mm, 2.6 μm) was used for HPLC. The column temperature was 35 °C, injection volume was 5 µL, and the flow rate was 0.3 mL/min. The mobile phases were shown as follows: positive mode A was 100% H_2_O (0.1% formic acid); positive mode B was 100% acetonitrile (0.1% formic acid); negative ion mode A was 100% H_2_O (0.5 mol/L NH4F); negative ion mode B was 100% supernatant pure water. The detection conditions for mass spectrometry included the use of an electrospray ion source (ESI) in cationic scanning and multi-reaction monitoring mode (MRM). The ion source temperature was maintained at 350 °C, with a drying air velocity of 9.0 L/min and atomizing gas pressure of 30 psi. Capillary voltage was set at 3.5 kV, breakage voltage was 0.1 kV, and cluster-removing voltage was 38 V. The collision voltage was 40 eV, and the detection dwell time was set to 100 s.

### 4.10. HPLC Analysis of ZEN Degradation

An overnight culture was divided into three aliquots, mixed thoroughly, and 10 µL was used to inoculate MM containing 10 μg/mL ZEN toxin as the experimental group. Pure MM without the culture broth (containing ZEN) was used as the control. After inoculation, the samples were incubated in a shaking incubator at 37 °C and 200 rpm for 12 h. At 4 h intervals, 0.5 mL of the culture solution was taken and combined with 0.5 mL of cold methanol. All samples were filtered through an organic filter membrane with a pore size of 0.22 µm. ZEN standard was dissolved in methanol, and the concentrations of ZEN standards were 0.3125, 0.625, 1.25, 2.5, 5, 10 and 20 µg/mL, respectively. Then, the concentration of ZEN was analyzed using HPLC (Agilent 1200, UV detector). As described in previous study [52], The HPLC consisted of an Agilent 1200 autosampler, an Agilent 1200 UV detector and an Agilent 1200 pump. An Agilent Eclipse XDB-C18 column (4.6 mm × 150 mm, 5 m) was used for chromatographic separation and the detection UV wavelength was 254 nm. The mobile phase was acetonitrile–water 60:40 (*v*/*v*) with a flow rate of 1 mL/min. the column was kept at 30 °C, and the injection volume was 20 μL. 

### 4.11. Statistical Analysis

All experiments were conducted at least in triplicate. The obtained data were analyzed using Origin v8.6 software (Origin Lab Corp., Northampton, MA, USA). The treatment groups were compared using one-way ANOVA, combined with the Student–Newman–Keuls method. Differences with *p*-values < 0.05 were considered statistically significant.

## Data Availability

Data is contained within the article or Supplementary Materials.

## Supplementary Materials

The following supporting information can be downloaded at: https://www.mdpi.com/article/10.3390/toxins15080488/s1, Figure S1: Effect of medium conditions on degradation of ZEN by B73 strain.
