# Supplementary material for: Isolation and Characterization of the Zearalenone-Degrading Strain, Bacillus spizizenii B73, Inspired by Esterase Activity
Source: Toxins (Basel). 2023 Aug 2;15(8):488. doi: 10.3390/toxins15080488 (PMC10467065; doi:10.3390/toxins15080488)
Supplement: Supplementary file 1 [file toxins-15-00488-s001.zip › toxins-2495958-SI.pdf]

Supplementary materials:
Isolation and Characterization of the Zearalenone-Degrading Strain, *Bacillus spizizenii* B73, Inspired by Esterase Activity

Xue Liu[1,†], Na Wu[2,†], Mingyu Zhang[1], Feng Xue [1,*] and Qing Xu [1,*]

[1] School of Food Science and Pharmaceutical Engineering, Nanjing Normal University, Nanjing 210046, China; liuxue@nnu.edu.cn (X.L.); mingyu@nnu.edu.cn (M.Z.)
[2] College of Marine and Bioengineering, Yancheng Institute of Technology, Yancheng 224007, China; nwu@cibt.ac.cn
* Correspondence: xuef2020@njnu.edu.cn (F.X.); xu_qing@njnu.edu.cn (Q.X.)
† These authors contributed equally to this work.

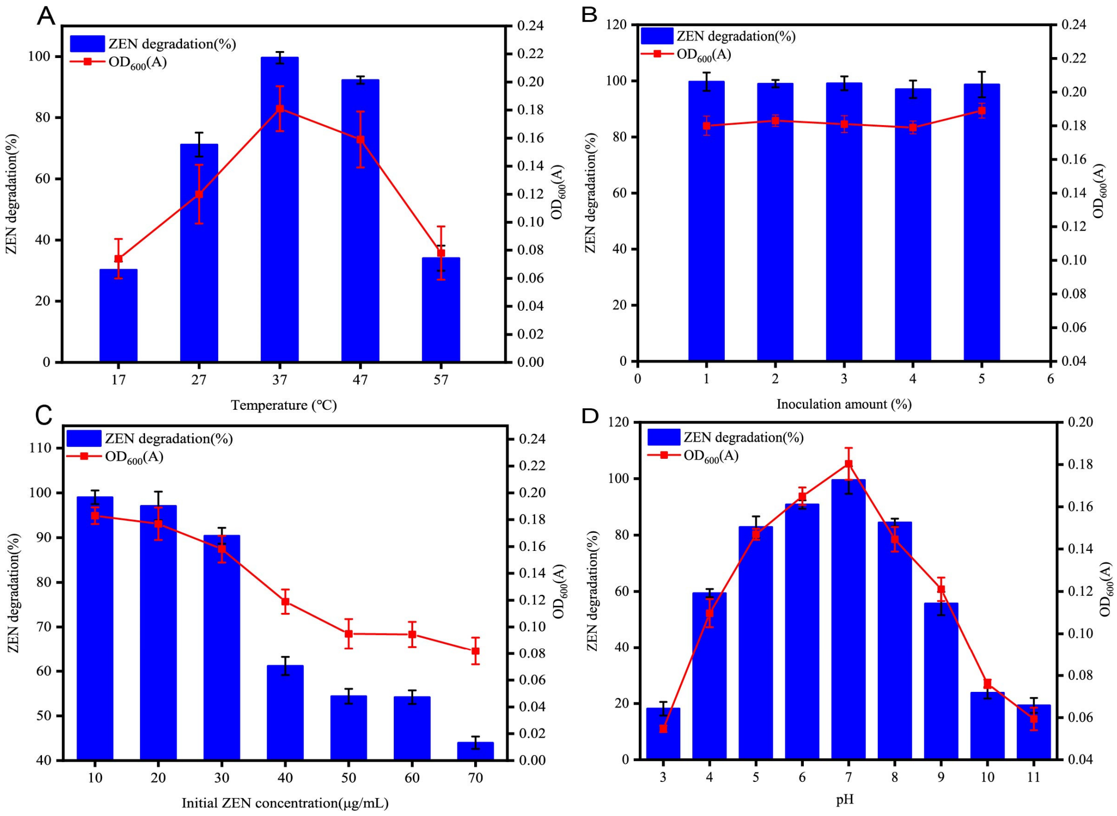

Figure S1. Effect of medium conditions on degradation of ZEN by B73 strain: (**A**) temperature, (**B**) inoculation amount, (**C**) initial ZEN concentration, and (**D**) pH.
